# Acute nicotine improves social decision-making in non-smoking but not in smoking schizophrenia patients

**DOI:** 10.3389/fnins.2013.00197

**Published:** 2013-10-30

**Authors:** Charel Quisenaerts, Manuel Morrens, Wouter Hulstijn, Peter de Boer, Maarten Timmers, B. Sabbe, Ellen R. A. de Bruijn

**Affiliations:** ^1^Collaborative Antwerp Psychiatric Research Institute, University of AntwerpAntwerp, Belgium; ^2^Psychiatric Hospital Broeders Alexianen BoechoutBoechout, Belgium; ^3^Donders Institute for Brain, Cognition, and Behaviour, Radboud University NijmegenNijmegen, Netherlands; ^4^Janssen Research and Development, a division of Janssen Pharmaceutica N.V.Beerse, Belgium; ^5^Psychiatric Hospital Sint-NorbertusDuffel, Belgium; ^6^Department of Clinical, Health and Neuropsychology, Leiden Institute for Brain and Cognition, Leiden UniversityLeiden, Netherlands

**Keywords:** nicotine, schizophrenia, ultimatum game, proactive control, fairness, intentionality

## Abstract

Schizophrenia patients are characterized by severe social impairments. Recently, social cognition has been put forward as an important mediator in schizophrenia between the often-reported neurocognitive deficits and functional outcome and is thus an important target for treatments. Nicotine has been reported to improve neurocognitive processes in schizophrenia patients but no studies have investigated possible nicotine-induced facilitation of social cognition. The current placebo-controlled crossover study aimed at bridging this gap by investigating whether the administration of active (1 mg or 2 mg) or placebo oromucosal nicotine spray resulted in improved social decision-making in non-smoking (*N* = 15) and smoking (*N* = 16) schizophrenia patients. All patients played the role of responder in a variant of the ultimatum game that allowed detailed measurements of fairness and intentionality considerations. The results showed impaired social decision-making in the non-smoking patients under placebo, but not in the smoking patients. Interestingly, this impairment normalized after administration of 1 mg of nicotine, but not after 2 mg of nicotine. Nicotine had no effect on performance in the smoking patients. The present study indicates that nicotine improves social decision-making in non-smoking patients. The present results suggest that acute nicotine effects may result in a facilitation of proactive control through improved attentional processes. However, the efficacy seems limited and although nicotine may thus be an interesting target for (social) cognitive enhancement in the subset of patients that do not smoke, more research is needed on the long-lasting effects of nicotine-based treatments.

## Introduction

Schizophrenia is a debilitating disorder, accompanied by severe impairments ranging from delusions and hallucinations to disorganized thinking, mood disturbances, and cognitive dysfunctions (Burton, 2006). In line with the evident problems in social behavior, more recent studies have demonstrated that patients with schizophrenia are also impaired in a wide range of social cognitive abilities, including emotion recognition (e.g., Kohler et al., 2010), mentalizing (e.g., Sprong et al., 2007; Harvey et al., 2013), and social decision-making (Csukly et al., 2011; Wischniewski and Brune, 2011). Cognitive dysfunctions, such as poor working memory performance, are known to be good predictors of functional outcome (Green et al., 2004). Interestingly, however, a recent meta analysis provided evidence for an important mediating role of social cognition in between neurocognitive deficits and functional outcome (Schmidt et al., 2011).

Since social cognitive deficits have tremendous effects on patients' wellbeing, their quality of life, as well as on functional outcome, there is an urgent need for methods to improve these processes in schizophrenia. One of the candidate components for cognitive enhancement is nicotine as both smoking and nicotine administration have been reported to improve sensory and attentional processes in humans (for a recent review see Heishman et al., 2010). In line with this, a growing number of studies have demonstrated nicotine to enhance neurocognitive processes in schizophrenia, such as sustained attention, anti-saccade performance, or delayed recognition (see e.g., Depatie et al., 2002; Myers et al., 2004; Barr et al., 2008). To this date, however, no one has investigated the effects of nicotine on social cognitive functioning in schizophrenia.

The aim of the present study is to bridge this gap by investigating effects of acute nicotine administration on social decision-making using a task derived from economic game theory in both non-smoking and smoking patients with schizophrenia. The main advantage of using economic games in studying social cognition is that they allow for the investigation of more real-life interpersonal interactions as compared to, for example, passive emotion-recognition paradigms. An often-used economic game is the so-called ultimatum game (UG; Güth et al., 1982) in which two players have to decide how to split a certain amount of money. One person proposes a split and the other person decides whether to accept or reject the offer. When the second person accepts the offer, the money is split as proposed, but when the decision is to reject both players receive nothing. Variations of the game allow for detailed measurements of processes such as fairness considerations, intentionality, and perspective taking (Falk et al., 2003). By limiting the number of offers the proposer can select from on each trial to two, one can systematically study the influence of the unchosen alternative on the decision-making process. Previous studies using this variant of the UG have demonstrated that unfair offers, for example, are more often rejected when they are paired with a fair alternative, thus demonstrating that humans incorporate fairness considerations. On the other hand, when an unfair is offer is paired with a similar and thus equally unfair offer, rejection rates decrease. Participants understand that in these cases the proposer had no choice and did thus not select the unfair offer intentionally. This latter process requires taking the perspective of the other into account and developmental studies using this variant of the game have shown for example, that the process of perspective taking is not fully developed until the age of 12 (Güroğlu et al., 2009). Studies using the UG have recently demonstrated deviant behavioral strategies and impaired social decision-making in schizophrenia patients (Csukly et al., 2011; Wischniewski and Brune, 2011).

Because of the previously demonstrated positive effects of nicotine on neurocognitive functioning, acute nicotine administration is expected to facilitate social decision-making in patients with schizophrenia. This hypothesis is supported by a recent meta-analysis by Ventura et al. (2013) demonstrating a close relationship between neurocognition and social-cognitive performance. Neurocognitive processes such as working memory, speed of processing, and attention showed moderate and relatively consistent relationships with the social-cognitive processes of emotion perception, social perception, and theory of mind. In line with this, a recent study by Fanning et al. (2012) revealed that normal range neurocognition is necessary, but not sufficient, for good social performance. It is expected that under the influence of nicotine, integration of different sources of information, such as fairness and intentionality should be enhanced. Recent research has demonstrated different nicotine-induced cortical excitability patterns between chronic and acute use in humans (Grundey et al., 2013). These differences in excitability may explain why Newhouse et al. (2011) reported task-related activity increases in non-smokers but not active smokers. Based on these recent findings, the enhancement was especially expected for the non-smoking patients with schizophrenia.

## Material and methods

### Participants

Two patient groups (16 smoking and 16 non-smoking patients with schizophrenia) were initially included in the study. One patient of the non-smoking group did not perform the task on one of the testing days and was therefore excluded from all analyses. Demographic and clinical characteristics of the final two groups are shown in Table 1. All patients were recruited from the Psychiatric hospital Sint-Norbertushuis in Duffel and the Psychiatric Hospital Stuivenberg in Antwerp, Belgium. All patients received some form of psychotherapy, such as cognitive-behavioral therapy. Importantly, none of the patients received specific social-cognitive therapy or social skills training. Of the final 15 participants in the non-smoking group, 11 patients were outpatients. In the smoking group, 9 of the 16 patients were outpatients. The study was carried out in correspondence with the latest version of the Helsinki Declaration and was approved by the medical–ethical committees of the participating hospitals. All participants gave their written informed consent. The study was performed according to Good Clinical Practice (GCP) and approved by Belgian Health Authority (HA). The clinicaltrials.gov identifier of this study is NCT01186471 and the EudraCT number is 2009-010616-14.

**Table 1:** **Demographic variables of smoking and non-smoking schizophrenic participants**.

	**Smoking (n = 16)**	**Non-smoking (n = 15)**	**Significance level of test** ^*^
Age (years)	32.6 (8.8)	40.1 (9.7)	0.031
Sex (M/F)	15/1	9/6	0.054
Education (years)	13.4 (2.3)	12.3 (2.6)	0.188
Chlorpromazine equivalent	403.9 (271.9)	303.8 (207.4)	0.261
Equivalent for males only	417.5 (192.2)	402.4 (275.8)	0.887
Equivalent for females only	200.0 (−)	155.8 (132.5)	–
Total PANSS score	61.4 (9.5)^^^	57.1 (9.4)	0.226
PANSS negative symptoms	16.8 (5.3)^^^	13.6 (2.9)	0.066
PANSS positive symptoms	12.7 (3.0)^^^	11.6 (3.1)	0.216
Cigarettes/day	24.6 (5.8)	/	/

Standard deviations are presented in parentheses.

* T-test for parametric data, Mann-Whitney test for non-parametric data.

^ PANSS scores were available for 15 patients in the Smoking group.

Smoking patients have been smoking at least 15 cigarettes/day for a minimum of 3 years. Any smoking cessation agent, such as nicotine replacement therapy, varenicline, or bupropion was not allowed during the period of the study. Non-smoking participants were excluded if they used cigarettes or other nicotine-based products within 3 months prior to the first experimental session. All patients were prescribed a stable dose of antipsychotic medication for at least two months prior to study assessments. Patients taking anti-depressive or mood-stabilizing medication in the two weeks prior to inclusion or anti-cholinergics two months prior to the first experimental session were excluded. One non-smoking patient taking a stable dose of a benzodiazepine for sleeping (lormetazepam 2 mg) was included in the experiment. Patients with a DSM-IV axis I diagnosis other than schizophrenia that had been the focus of treatment or cause of disability in the last 6 months, were excluded. Also, substance abuse or dependence (e.g., cannabis, alcohol) other than nicotine was an exclusion criterion for all participants. Finally, participants with a history of or current significant medical disease were excluded as well.

Screening examination consisted of laboratory (serology, hematology, chemistry, toxicology, urinalysis, and serum pregnancy test for women of childbearing potential), ECG and clinical investigations by the research physician. Any clinical significant deviation in these assessments was an exclusion criterion.

### Design

The study was a double-blind placebo-controlled randomized three-way crossover design where participants received active (1mg or 2mg) or placebo oromucosal nicotine spray (Nicorette mouth spray 1mg/dose), which was granted for free by McNeil AB, Sweden. The placebo spray was identical in appearance and formulation, except for nicotine, which was replaced with capsaicin to mimic the taste of nicotine. The experimental design consisted of three separate test sessions 2 to 7 days apart, sessions in which participants received one of the three conditions in a counterbalanced order. Fourteen neurocognitive tasks were administered each testing day, distributed over three test blocks of which the order was counterbalanced between participants and experimental sessions. The results of the other cognitive tasks will be reported separately. The duration of the different test blocks was 45–60 min and the interval between the test blocks lasted 150 min. The participants received their dosage prior to each test block (i.e., 30 min before task onset), three times a day in total. Smokers were not allowed to smoke 2h pre-dose. After each test block smokers were allowed to smoke a maximum of three cigarettes in a 15 min period to prevent possible effect of nicotine withdrawal on the assessments. Blood samples were taken at three time points relative to each administration (pre-dose, 5 min post-dose, and 1h post-dose, for mean values see Table 2).

**Table 2:** **Mean plasma concentration nicotine (ng/ml)**.

**COHORTS**	**Dose**	**FIRST INTAKE**			**SECOND INTAKE**			**THIRD INTAKE**		
		**PREDOSE**	**5 MIN**	**60 MIN**	**PREDOSE**	**5 MIN**	**60 MIN**	**PREDOSE**	**5 MIN**	**60 MIN**
Non smoking patients	0 mg	BQL	BQL	BQL	BQL	BQL	BQL	BQL	BQL	BQL
	1 mg	BQL	1.9 (1.3)	1.4 (0.6)	1.0 (0.3)	3.5 (2.3)	2.4 (0.7)	1.3 (0.5)	3.3 (0.9)	2.4 (0.7)
	2 mg	BQL	3.4 (2.8)	2.6 (1.3)	1.9 (0.7)	4.7 (1.8)	4.4 (1.5)	2.6 (0.8)	6.7 (2.9)	4.6 (1.5)
Smoking patients	0 mg	12.6 (6.8)	11.7 (6.5)	8.4 (4.8)	15.7 (7.1)	13.1 (6.1)	9.8 (4.7)	15.3 (7.4)	13.2 (6.6)	9.6 (4.8)
	1 mg	11.6 (6.5)	11.6 (6.5)	8.9 (4.2)	13.4 (6.7)	13.1 (5.9)	10.3 (4.4)	12.9 (4.5)	13.7 (4.3)	10.4 (3.6)
	2 mg	11.8 (7.3)	12.5 (5.9)	10.2 (4.0)	15.3 (5.3)	16.0 (5.1)	12.7 (3.6)	14.9 (3.9)	16.7 (3.9)	14.3 (2.7)

Standard deviations are presented in parentheses.

BQL = Below Quantification Limit / Smokers: n = 15 / Non-smokers: n = 15 (2 mg) and n = 14 (1 mg and placebo).

### Task

**Figure 1:**
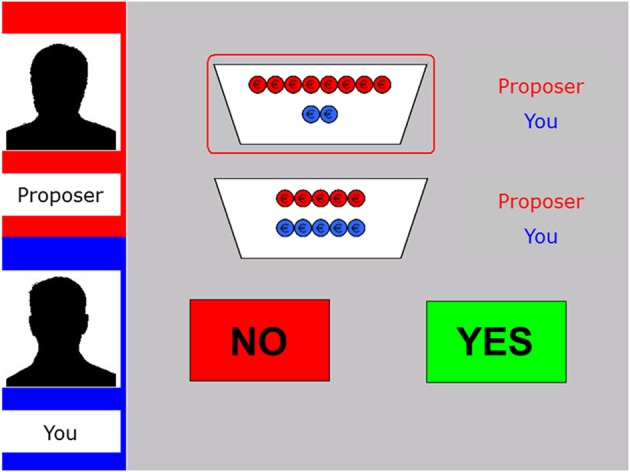
**Display of the decision phase in the fair-alternative condition of the modified UG**. The left panel shows the name and silhouette of the proposer at the top (here “Proposer”) as well as the name of the participant underneath (here “You”). The two potential distributions are specified by red and blue coins (red for the proposer, blue for the responder; here 8:2 vs. 5:5). The selected offer is encircled in red. The participant has to decide whether to accept (“Yes”) or reject (“No”) the offer via button press.

The mini Ultimatum Game (mini-UG; Falk et al., 2003; Güroğlu et al., 2009) was computerized and programmed in E-prime version 2. In this version of the UG, a sum of money (€10) is split in different distributions, named from the perspective of the proposer: a fair one (5:5; 5 for the proposer, 5 for the responder), a hyperfair one (2:8; 2 for the proposer, 8 for the responder), an unfair distribution (8:2; 8 for the proposer, 2 for the responder), or a hyperunfair one (10:0; 10 for the proposer, 0 for the responder). On each trial, the computer selects two possible distributions (e.g., a hyperfair vs. an unfair distrubtion: 2:8 vs. 8:2) of which the proposer has to choose one (see Figure 1).

Next, the responder can accept or reject the proposed offer, by pressing one of two buttons. If the responder accepts, both players earn the amount of money as specified in the proposal. Importantly, however, if the responder rejects, both players receive nothing. All participants played the role of the responder and were led to belief that the choices of the proposers were based on behavioral data from subjects who had previously participated as proposers. Furthermore, patients were instructed that they played every round with a new proposer.

In total, 48 pairs of offers were presented by the computer (12 unfair-fair, 12 unfair-hyperfair, 12 unfair-hyperunfair and 12 unfair-unfair). The software is programmed such that on 30 trials (6 unfair-fair, 6 unfair-hyperfair, 6 unfair-hyperunfair and 12 unfair-unfair) the proposer selects the unfair offer out of the two available distributions. The unfair-unfair distribution is referred to as the “no alternative” condition, as the proposer does not have a real alternative choice. The primary outcome measure was the rejection rates of the responder to the proposed unfair offers in the four alternative conditions: alternative offer is (1) unfair, (2) fair, (3) hyperfair, and (4) hyperunfair. The alternative conditions are also referred to as the ‘context'. On the remaining 18 trials, the proposer selects 6 fair, 6 hyperfair and 6 hyperunfair offers instead of the unfair alternative. These data were not used in the main analyses, but served as filler items.

This version of the UG has been constructed to investigate the participants' capability to make social decisions on the basis of fairness and intentionality considerations in different contexts. For example, higher rejection rates are expected when an unfair offer is selected over a fair alternative, compared to when the alternative was a hyperunfair offer. Also, lower rejection rates are expected in the no alternative condition, when the proposer has no other choice than choosing an unfair offer and thus not treats the responder unfair in an intentional manner (see e.g., Güroğlu et al., 2009; Radke and de Bruijn, 2012; Radke et al., 2012).

To strengthen the concept of an interactive game, it was emphasized to participants that their decisions also affected the other players' outcome, because the eventual payoff of the proposers would be determined by their response and proposers would be paid after all data from the responders had been collected. Furthermore, participants were informed that at the end of the experiment a random number of rounds would be selected to determine the payoff. This was done to ensure participants' motivation and perception of a one-shot game, as every trial could influence their financial outcome. The payoff was set at 10 euro over the three periods. The total duration of the task was 15–20 min.

### Statistical analyses

Analyses were run in SPSS Statistics version 20.0. Rejection rates of unfair offers were entered into GLM repeated measures ANOVAs with Group (two level: smoking vs. non-smoking patients) as between-subjects factor and Dose (three levels: 0 mg vs. 1 mg vs. 2 mg) and Context (four levels: no-alternative vs. fair vs. hyperfair vs. hyperunfair) as within-subject factors. As analyses of the demographic variables revealed a significant difference in age between the two groups (see Table 1), age was entered as a covariate in the analyses. In accordance with previous studies using the same paradigm (e.g., Güroğlu et al., 2009; Radke et al., 2012), a significance level of 0.05 was applied for all analyses and significant interactions were followed up by ANOVAs or two-tailed t-tests. Greenhouse–Geisser corrections were applied when appropriate, but uncorrected degrees of freedom values are given for ease of interpretation.

## Results

### Overall analyses of rejection rates

**Figure 2:**
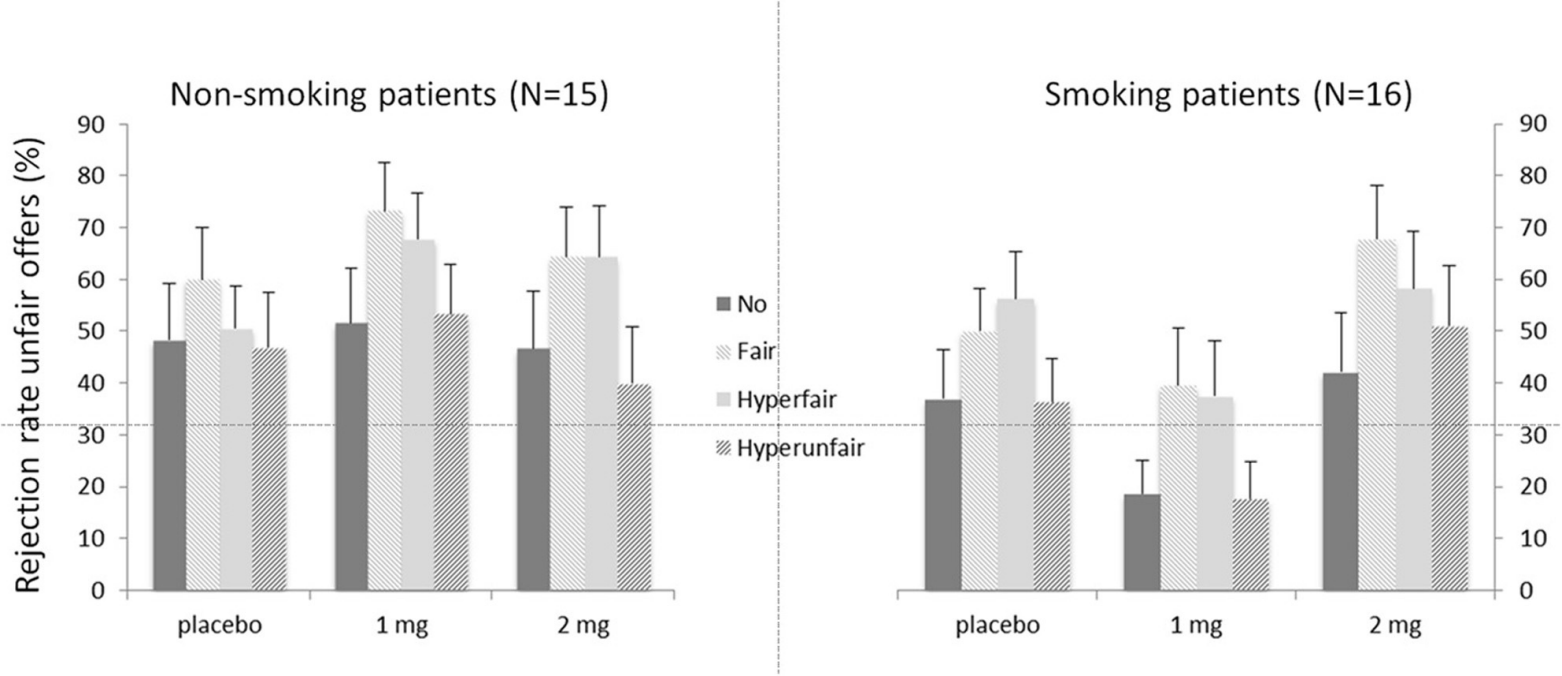
**Mean rejection rates in percentages for unfair offers depending on the alternative offers (context) for the two patient groups**. Error bars represent standard errors of the mean.

Figure 2 depicts the rejection rates for the different offers (i.e., Context) for the two patient groups. As expected, a main effect of Context was present [*F*_(3, 26)_ = 3.05, *p* = 0.047], indicating that rejection rates were dependent on the unchosen alternative offer. Neither the main effect of Group [*F*_(1, 28)_ = 1.47, *p* = 0.236], nor the main effect of Dose was significant [*F*_(2, 27)_ = 1.93, *p* = 0.165]. The interaction between Dose and Context [*F*_(6, 23)_ = 2.60, *p* = 0.045] was significant. Neither the interaction between Context and Group [*F* < 1], nor the interaction between Dose and Group [*F*_(2, 27)_ = 2.94, *p* = 0.070] was significant. None of the interactions with the covariate age were significant [all *F*s < 1.95, all *p*s > 0.146], except for the three-way interaction between Dose, Context and Age [*F*_(6, 23)_ = 3.38, *p* = 0.016]. Importantly, the three-way interaction between Dose, Context, and Group was significant [*F*_(6, 23)_ = 3.45, *p* = 0.014]. The latter interaction was further investigated by analyzing the two groups separately for effects of Dose and Context.

#### Smoking schizophrenia patients

The main effect of Context was significant [*F*_(3, 13)_ = 6.32, *p* = 0.007]. Neither the main effect of Dose [*F*_(2, 14)_ = 2.95, *p* = 0.085], nor the interaction between Dose and Context was significant [*F* < 1]. Follow-up contrasts for Context showed the expected pattern, with increased rejections for fair (52.4%; *p* = 0.001) and hyperfair (50.7%; *p* = 0.008) alternatives compared to the no-alternative condition (32.6%). The hyperunfair condition (35.1%) did not differ from the no-alternative one (*p* = 0.473).

#### Non-smoking schizophrenia patients

For the non-smoking patient group, the main effect of Context was not significant [*F*_(3, 13)_ = 2.06, *p* = 0.16]. The main effect of Dose was marginally significant [*F*_(2, 14)_ = 3.54, *p* = 0.059]. Importantly, the interaction between Context and Dose was significant [*F*_(6, 10)_ = 4.07, *p* = 0.030]. This interaction was caused by the finding that the non-smoking patients failed to show an effect of Context in the placebo condition [*F* < 1]. They did however show a main effect of Context after 1 mg of nicotine [*F*_(3, 12)_ = 4.85, *p* = 0.015], but only a trend was seen after 2 mg of nicotine [*F*_(3, 12)_ = 2.97, *p* = 0.074]. After 1 mg of nicotine, the expected pattern was found, with increased rejection rates for fair alternatives (73.3%) compared to no-alternatives [51.6%; *p* = 0.017]. The difference between the hyperfair (67.8%) and no-alternative condition was marginally significant [*p* = 0.056] and the hyperunfair (53.3%) alternative did not differ from the no-alternative [*p* = 0.731].

## Discussion

The aim of the current study was to investigate the effects of nicotine on social decision-making in smoking and non-smoking schizophrenia patients. As demonstrated repeatedly in healthy volunteers, the smoking patients' decisions were affected by the unchosen alternative offer in the expected pattern (see e.g., Güroğlu et al., 2009; Radke and de Bruijn, 2012; Radke et al., 2012). In other words, smoking patients incorporated both fairness and intentionality considerations into their social decision-making process. The non-smoking patients, however, failed to take these considerations into account under placebo. Interestingly, after administration of 1mg of nicotine, the non-smoking patients also showed the similar decision pattern incorporating fairness and intentionality considerations. The smoking patients' responder behavior was not affected by nicotine.

The current outcomes may be explained by impairments in a mechanism that was recently put forward by Barch and Ceaser (2012). The authors propose a common mechanism driving the variety of deficits seen in schizophrenia, i.e., an impaired ability to actively represent goal information in working memory to guide behavior. This so-called proactive control heavily relies on dorsolateral prefrontal cortex (DLPFC) and impairments in this brain area or connected areas may thus importantly result in problems in this central function. DLPFC dysfunctions have often been implicated in schizophrenia, for example reduced activations in schizophrenia patients have been repeatedly demonstrated during working memory tasks (see e.g., Meyer-Lindenberg et al., 2005; Barch and Csernansky, 2007).

Importantly, proactive control is also central for adequate social decision-making, as it requires maintaining a representation of the goal and integrating it in a complex context involving elements of fairness and intentionality. For example, the unchosen alternative offer has to remain active in working memory to influence the final decision-making process (“What I have” versus “What I could have had”). Also, one needs to take the perspective of the other person into account in order to fully understand if an unfair offer was made intentionally (fair alternative) or unintentionally (no alternative). This process requires assessment of cognitive perspective taking and a recent developmental study using this version of the UG has demonstrated that typically developing children do not take alternative offers into account until the age of 12 (Güroğlu et al., 2009). This finding is in line with the protracted development of the involved brain regions such as DLPFC (Crone and Dahl, 2012) and with the idea that perspective taking is a function that develops slowly and well into late adolescence (Dumontheil et al., 2010) and may thus be a particularly vulnerable process in schizophrenia.

Although we are obviously careful in drawing conclusions regarding neural mechanisms based on behavioral data alone, fMRI studies using the UG have repeatedly demonstrated an important role for DLPFC in response to unfair offers. The area seems to play a central role in the implementation of fairness motives when self-interests are competing (Sanfey et al., 2003; Güroğlu et al., 2011). Also, disruption of DLPFC using transcranial magnetic stimulation (TMS) resulted in increased acceptance rates of unfair offers (van't Wout et al., 2005; Knoch et al., 2006), implicating that following TMS participants did not implement their fairness goals in the social-decision-making process.

The current findings may thus indicate that nicotine improves proactive control in the non-smoking schizophrenia patients. This interpretation is supported by neuroimaging findings that reveal nicotine-induced activations in these tasks mainly in the prefrontal cortex (for a recent review see Newhouse et al., 2011). After administration of nicotine, non-smoking schizophrenia patients may implement their fairness goals more in the decision-making process, possibly through enhancement of DLPFC activation. Thus, in non-smoking patients with schizophrenia, a deficient cholinergic system (see e.g., Leonard et al., 1998, 2007; Mexal et al., 2010) in combination with diminished DLPFC activity (Meyer-Lindenberg et al., 2005; Barch and Csernansky, 2007) may create the fundament for possible nicotine-induced stimulation of proactive control (Barch and Ceaser, 2012). Obviously, dedicated neuroimaging studies are needed to test the latter hypothesis in the near future. Such studies would also shed more light on whether improved proactive control results from a direct influence on DLPFC or from a more indirect pathway through facilitation of low-level processes. In support of the latter interpretation, previous studies have demonstrated effects of nicotine on processes required for proactive control, such as sustained attention (for a recent meta-analysis see Heishman et al., 2010). Thus improved social decision-making in the current paradigm may also result of more low-level enhancement of attentional processes. For example, non-smoking patients may attend more to the context after nicotine administration. This may have an effect on the decisional value of the contextual differences and may thus result in the currently found response patterns. An interesting way to specifically investigate this issue in the near future is by making use of eye-movement measurements during performance of the UG. By doing so, one could easily establish whether patients attend to the context in the first place and whether this attention can be modulated by nicotine administration.

Interestingly, the optimum in non-smoking patients is reached after 1 mg nicotine with a slight decline after 2 mg, suggestive for saturated nicotine concentrations in non-smoking patients after 1mg oromucosal nicotine spray. These findings are in line with a model of nicotinergic stimulation by Newhouse et al. (2004), in which the authors propose the dose-response curve to follow an inverted U shape (see e.g., Perkins et al., 1994; Poltavski et al., 2012). This means that stimulation by nicotine initially improves cognitive performance and once the optimum is reached further stimulation is not effective or even detrimental. This interpretation is in line with the idea that patients with schizophrenia are characterized by hypocholinergic function and performance scores on the left side of the dose-response curve, thus making room for cognitive enhancement.

Smoking patients, on the other hand, did show integration of context irrespective of dosage and were not able to improve with nicotine. The most likely explanation is chronic saturation with nicotine in smoking patients, placing them more to the right on the inverted U curve. This was supported by the high plasma nicotine values at placebo and by the small additive effects of oromucosal nicotine spray on the plasma levels. Note that this finding is likely to be related to smoking patients being allowed to smoke in between the test blocks to avoid unwanted effects of withdrawal. Therefore, the current facilitating outcomes of nicotine for the non-smoking patients only are in line with Newhouse et al. (2011) who recently concluded that “nicotine appears to increase task-related activity in non-smokers and deprived smokers, but not active smokers.”

However, we would like to point out that based on the current results we cannot exclude an alternative interpretation stating that higher doses would have led to behavioral changes in this group. Visual inspection of Figure 2, for example, may suggest more similar response patterns after 2 mg in the smoking patients and after 1 mg of nicotine in the non-smoking group. This resemblance, combined with the small additive effects of nicotine administration on plasma levels in the smoking patients, could thus suggest that patients' lack of improvement may be due to too low nicotine doses. It is important to note though, that the absolute performance measures as reflected in these response patterns are less relevant for the current study. Our main interest lies in the presence of an effect of context, indicative of taking alternative unselected offers into account. Therefore, the relative influence of the different conditions rather than absolute rejection rates is crucial for the studied processes of fairness and perspective taking. Moreover, as smoking patients already show this distinction after placebo, it is not immediately evident how performance could further improve.

We would like to emphasize that the current results warrant replication, especially given the relatively small sample size. However, to our knowledge, this is the first study that investigated the effects of nicotine on social cognition in schizophrenia patients. Previous animal studies have repeatedly shown that nicotine administration leads to oxytocin release in rats (see e.g., Bisset and Walker, 1957; Russell and Chaudhury, 1972), probably due to the effect nicotine has on the oxytocinergic neurons in the hypothalamic paraventricular nuclei (Mikkelsen et al., 2012). Over the past years, oxytocin has received a lot of attention and a growing number of studies have revealed a central role for the hormone in a variety of human social cognitive processes, including social memory, emotion perception, empathy, and trust. Moreover, authors have also emphasized the possible potential of oxytocin for clinical populations (for recent reviews see e.g., Guastella and MacLeod, 2012; Zink and Meyer-Lindenberg, 2012; Bakermans-Kranenburg and van IJzendoorn, 2013). In line with this, two recent studies have demonstrated oxytocin-induced improvements in schizophrenia patients for social perception (Fischer-Shofty et al., 2013) and for high-level social cognitive processes, such as deception detection and empathy (Davis et al., 2013). However, translation of these findings into therapeutic applications warrants further investigation, especially since recent studies in healthy volunteers have also reported ‘antisocial' rather than prosocial effects (see e.g., De Dreu et al., 2010, 2011; Bartz et al., 2011; Radke et al., 2012, 2013). The current findings thus importantly add to the existing literature by showing that nicotine, known for its cognitive enhancing role, may also facilitate central aspects of social cognition necessary for efficient social behavior. An interesting question for future studies remains whether or not this nicotine-induced facilitation is mediated by the release of oxytocin.

To conclude, the current study showed improved social decision-making in non-smoking patients with schizophrenia after administration of 1 mg of nicotine. Although the present study shows that nicotine may facilitate social cognitive processes, it also suggests that the efficacy may be limited as the effects were no longer present after a higher dose and did not apply to the smoking patients. Thus, the possible clinical relevance of these findings remain unclear and more research is needed. For example, the effects of chronic nicotine administration are unknown and it is difficult to predict how exactly the currently found effects would translate to real-world settings. However, the presently demonstrated acute effects may make nicotine an interesting candidate as an adjunct to existing therapies, such as social-cognitive remediation. Future studies should investigate whether the efficacy of existing treatment programs is improved by the nicotine-induced facilitation of social decision-making processes. In short, the current findings demonstrate that nicotine may facilitate social cognitive processes through improved integration of different sources of information and may thus especially be an interesting target for cognitive enhancement in the subset of patients that do not smoke.

## Author contributions

Design study Ellen R. A. de Bruijn, Collecting data Charel Quisenaerts, Analyzing data (Charel Quisenaerts, Ellen R. A. de Bruijn), Writing first version (Charel Quisenaerts, Ellen R. A. de Bruijn), Providing feedback (Manuel Morrens, Wouter Hulstijn, Peter de Boer, Maarten Timmers, B. Sabbe), Finalizing manuscript (Charel Quisenaerts, Ellen R. A. de Bruijn).

### Conflict of interest statement

P. de Boer and M. Timmers are full-time employees of Janssen Pharmaceutica N.V. All other authors declare that they have no conflict of interest.
